# Supplementary material for: Mutations upstream from sdaC and malT in Escherichia coli uncover a complex interplay between the cAMP receptor protein and different sigma factors
Source: J Bacteriol. 2024 Jan 10;206(2):e00355-23. doi: 10.1128/jb.00355-23 (PMC10882989; doi:10.1128/jb.00355-23)
Supplement: Supplemental material — Figures S1 to S3; Tables S1 and S2. [file jb.00355-23-s0001.docx]

Supplementary materials for

**Mutations upstream from *sdaC* and *malT* in *Escherichia coli* uncover a complex interplay between the cAMP receptor protein and different sigma factors**

**Data availability.** Source data for this paper are available from the authors.


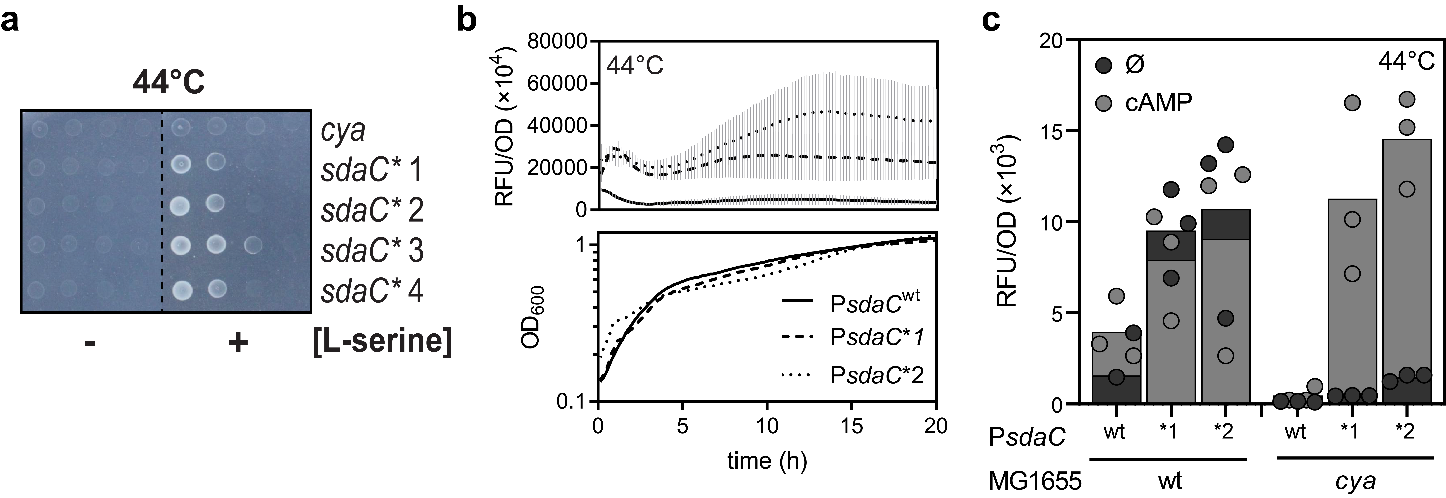
 **Supplementary Figure 1.** Effects of P*sdaC** at 44*°*C. a) Serial dilutions of *E. coli* K-12 MG1655 *cya* with the native or one of two promoter mutations in P*sdaC*, on M9 minimal media at 37*°*C in the presence or absence of serine as a carbon source. Two mutants of each mutation type were found and are marked (1) and (2). b) Promoter activity for the native (wt) and mutant (*) *sdaC* promoters in *E. coli* K-12 wild-type and *cya* after 20 h of growth at 44*°*C. For each strain-reporter combination, two growth conditions are superimposed; growth with no supplement (Ø, dark grey) or in the presence of 0.5 mM cAMP (grey). c) Fluorescence from the native (wt) and mutant (*) *sdaC* promoters in *E. coli* K-12 wild-type and *cya* after 12 h of growth at 44*°*C. For each strain-reporter combination, two growth conditions are superimposed (not staggered); growth with no supplement (Ø, dark grey) or in the presence of 0.5 mM cAMP (grey). The tallest bar is shown in the back, and the shortest in front.


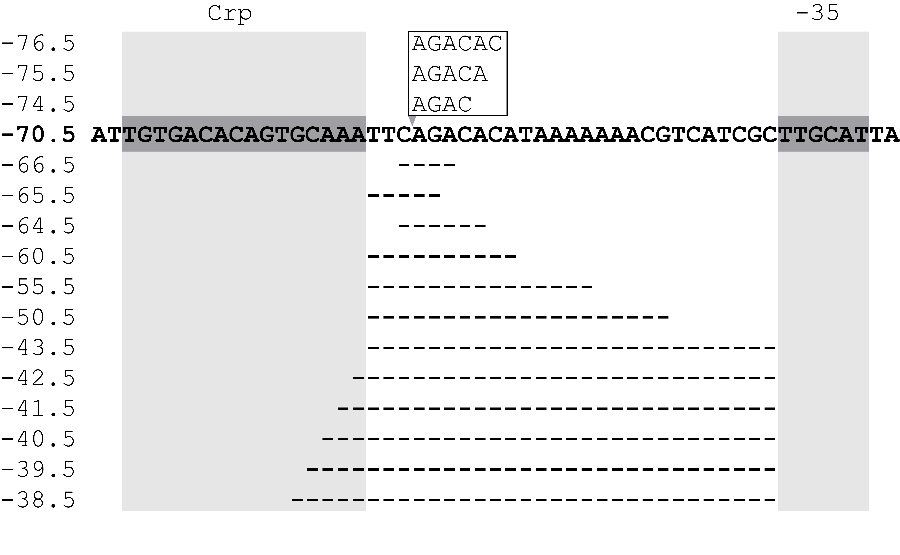


**Supplementary Figure 2.** The native *malT* promoter and the Crp distance mutants investigated in this study.


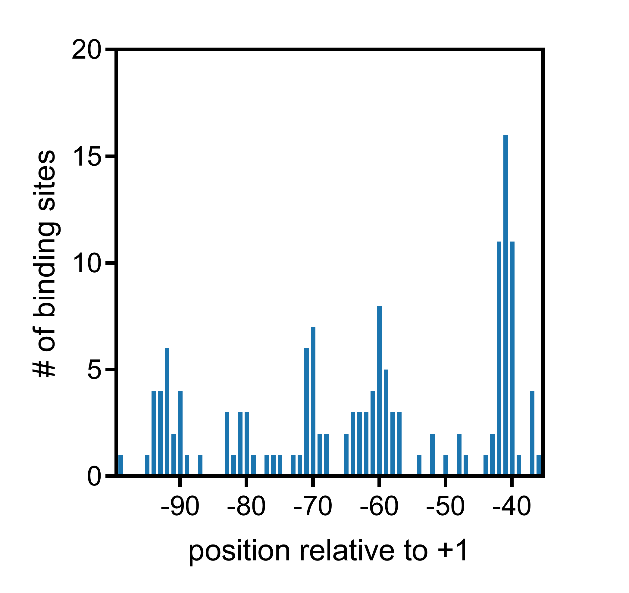


**Supplementary Figure 3.** Distribution of activating Crp binding sites on RpoD promoters. Data from RegulonDB, 26-11-2021 (Santos-Zavaleta 2019).

**Supplementary Table 1.** Strains and plasmids used in this study.

| Strain | Genotype | Reference |
| --- | --- | --- |
| WT | K-12 MG1655 WT | Our laboratory |
| *rpoS* | K-12 MG1655 Δ*rpoS* | A. Ehrmann |
| *cya* | K-12 MG1655 *cyaA::cat* Δ*fnr* | Sekowska 2016 |
| *crp** | K-12 MG1655 *cyaA::cat* ∆*fnr crp*A144T | Sekowska 2016 |
| *rpoS*N98K | K-12 MG1655 *cyaA::cat* ∆*fnr crp*A144E *rpoS*N98K | Sekowska 2016 |
| *sdaC**1 (1) | K-12 MG1655 *cyaA::cat* ∆*fnr sdaC**1 | Heyde 2021 |
| *sdaC**1 (2) | K-12 MG1655 *cyaA::cat* ∆*fnr sdaC**1 | Heyde 2021 |
| *sdaC**2 (1) | K-12 MG1655 *cyaA::cat* ∆*fnr sdaC**2 | Heyde 2021 |
| *sdaC**2 (2) | K-12 MG1655 *cyaA::cat* ∆*fnr sdaC**2 | Heyde 2021 |
| *cya crp* | K-12 MG1655 *cyaA::cat* ∆*fnr* ∆*crp*[5](#_bookmark673) |  |
| *cya crp rpoS* | K-12 MG1655 *cyaA::cat* ∆*fnr* ∆*crp* ∆*rpoS* | I. Lauritsen |
| *cya crp rpoS*N98K | K-12 MG1655 *cyaA::cat* ∆*fnr* ∆*crp* ∆*rpoS*N98K | I. Lauritsen |
| Plasmids |  | Description |
| pSEVA27-*crp* | *crp* expression from native *crp* promoter, | Lauritsen 2021 |
| pGEM-P*malT*-hp- sfGFP_noKan | *sfgfp-ssrA*-pHP14 hairpin, expression from *malT* promoter, strong SD, ColE1 replicon, AmpR | Lauritsen 2021 |
| pGEM-P*malT**- hp-sfGFP_noKan | *sfgfp-ssrA*-pHP14 hairpin, expression from modified *malT* promoter, strong SD, ColE1  replicon, AmpR | Lauritsen 2021 |
| pGEM-P*malT*(-10)- hp-sfGFP_noKan | *sfgfp-ssrA*-pHP14 hairpin, expression from modified *malT* promoter, strong SD, ColE1  replicon, AmpR | This study |
| pGEM-P*malT*(-35)- hp-sfGFP_noKan | *sfgfp-ssrA*-pHP14 hairpin, expression from modified *malT* promoter, strong SD, ColE1  replicon, AmpR | This study |
| pGEM-P*malT*(-10/-35)- hp-sfGFP_noKan | *sfgfp-ssrA*-pHP14 hairpin, expression from modified *malT* promoter, strong SD, ColE1  replicon, AmpR | This study |
| pGEM-P*malT*(-76.5)- hp-sfGFP_noKan | *sfgfp-ssrA*-pHP14 hairpin, expression from modified *malT* promoter, strong SD, ColE1  replicon, AmpR | This study |
| pGEM-P*malT*(-75.5)- hp-sfGFP_noKan | *sfgfp-ssrA*-pHP14 hairpin, expression from modified *malT* promoter, strong SD, ColE1  replicon, AmpR | This study |
| pGEM-P*malT*(-74.5)- hp-sfGFP_noKan | *sfgfp-ssrA*-pHP14 hairpin, expression from modified *malT* promoter, strong SD, ColE1  replicon, AmpR | This study |
| pGEM-P*malT*(-66.5)- hp-sfGFP_noKan | *sfgfp-ssrA*-pHP14 hairpin, expression from modified *malT* promoter, strong SD, ColE1  replicon, AmpR | This study |
| pGEM-P*malT*(-65.5)- hp-sfGFP_noKan | *sfgfp-ssrA*-pHP14 hairpin, expression from modified *malT* promoter, strong SD, ColE1  replicon, AmpR | This study |
| pGEM-P*malT*(-64.5)- hp-sfGFP_noKan | *sfgfp-ssrA*-pHP14 hairpin, expression from modified *malT* promoter, strong SD, ColE1  replicon, AmpR | This study |
| pGEM-P*malT*(-60.5)- hp-sfGFP_noKan | *sfgfp-ssrA*-pHP14 hairpin, expression from modified *malT* promoter, strong SD, ColE1  replicon, AmpR | This study |
| pGEM-P*malT*(-55.5)- hp-sfGFP_noKan | *sfgfp-ssrA*-pHP14 hairpin, expression from modified *malT* promoter, strong SD, ColE1  replicon, AmpR | This study |
| pGEM-P*malT*(-50.5)- hp-sfGFP_noKan | *sfgfp-ssrA*-pHP14 hairpin, expression from modified *malT* promoter, strong SD, ColE1  replicon, AmpR | This study |
| pGEM-P*malT*(-43.5)- hp-sfGFP_noKan | *sfgfp-ssrA*-pHP14 hairpin, expression from modified *malT* promoter, strong SD, ColE1  replicon, AmpR | This study |
| pGEM-P*malT*(-42.5)- hp-sfGFP_noKan | *sfgfp-ssrA*-pHP14 hairpin, expression from modified *malT* promoter, strong SD, ColE1  replicon, AmpR | This study |
| pGEM-P*malT*(-42.5)- hp-sfGFP_noKan | *sfgfp-ssrA*-pHP14 hairpin, expression from modified *malT* promoter, strong SD, ColE1  replicon, AmpR | This study |
| pGEM-P*malT*(-40.5)- hp-sfGFP_noKan | *sfgfp-ssrA*-pHP14 hairpin, expression from modified *malT* promoter, strong SD, ColE1  replicon, AmpR | This study |
| pGEM-P*malT*(-39.5)- hp-sfGFP_noKan | *sfgfp-ssrA*-pHP14 hairpin, expression from modified *malT* promoter, strong SD, ColE1  replicon, AmpR | This study |
| pGEM-P*malT*(-38.5)- hp-sfGFP_noKan | *sfgfp-ssrA*-pHP14 hairpin, expression from modified *malT* promoter, strong SD, ColE1  replicon, AmpR | This study |
| pGEM-P*sdaC*- hp-sfGFP_noKan | *sfgfp-ssrA*-pHP14 hairpin, expression from *sdaC* promoter, strong SD, ColE1  replicon, AmpR | This study |
| pGEM-P*sdaC**1- hp-sfGFP_noKan | *sfgfp-ssrA*-pHP14 hairpin, expression from modified *sdaC* promoter, strong SD, ColE1  replicon, AmpR | This study |
| pGEM-P*sdaC**2- hp-sfGFP_noKan | *sfgfp-ssrA*-pHP14 hairpin, expression from modified *sdaC* promoter, strong SD, ColE1  replicon, AmpR | This study |

**Supplementary Table 2.** Oligonucleotides used in this study.

| Oligo | Name | Sequence (5’- >3’) |
| --- | --- | --- |
| 4256 | pGEM_PmalT_rv | AATCCCAATUCACTGGCCGTCGTTTTAC |
| 4258 | php14-RBS-sfGFP_fw | AGTGAGATUGTTGACGGTACCGTATTTTCCTCTAGAAATAA TTTTGTTTAACTTTAAGAAGGAGATATACCATGAGCAAAGG CGAAGAGCTGTTCACTG |
| 4577 | USER_PsdaC_fw | AATTGGGATUGCCGGGTTTTCTCGTTTTTG |
| 4578 | USER_PsdaC_rv | AATCTCACUCGAGAGTCGACGTGAGGAATGCAAATAATTC AGCTTTTAGCC |
| 5590 | PmalT_dist_variants_fw | AGAAAGGTUTCTGGCCGACC |
| 5591 | PmalT_CRP-65_rv | AACCTTTCUAATGCAAGCGATGACGTTTTTTTATGTGTTTT GCACTGTGTCACAATTCCAAATC |
| 5592 | PmalT_CRP-60_rv | AACCTTTCUAATGCAAGCGATGACGTTTTTTTATTTGCACT GTGTCACAATTCCAAATC |
| 5593 | PmalT_CRP-55_rv | AACCTTTCUAATGCAAGCGATGACGTTTTTTGCACTGTGTC ACAATTCCAAATC |
| 5594 | PmalT_CRP-50_rv | AACCTTTCUAATGCAAGCGATGATTTGCACTGTGTCACAAT TCCAAATC |
| 5595 | PmalT_CRP-43_rv | AACCTTTCUAATGCAATTTGCACTGTGTCACAATTCCAAA TC |
| 5596 | PmalT_CRP-42_rv | AACCTTTCUAATGCAATTGCACTGTGTCACAATTCCAAATC |
| 5597 | PmalT_CRP-41_rv | AACCTTTCUAATGCAATGCACTGTGTCACAATTCCAAATC |
| 5598 | PmalT_CRP-40_rv | AACCTTTCUAATGCAAGCACTGTGTCACAATTCCAAATC |
| 5599 | PmalT_CRP-39_rv | AACCTTTCUAATGCAACACTGTGTCACAATTCCAAATC |
| 5600 | PmalT_CRP-38_rv | AACCTTTCUAATGCAAACTGTGTCACAATTCCAAATC |
| 5601 | PmalT_-35_variants_fw | ATTAATACGUCGACTCTCGAGTGAGATTGTTG |
| 5726 | PmalT_CRPdist0_rv | ATGACGTTUTTTTATGTGTCTGAATTTGCACTGTGTCACA ATTCC |
| 5727 | PmalT_CRPdist-4_rv | ATGACGTTUTTTTATGTGTCTGTCTGAATTTGCACTGTGT CACAATTCC |
| 5728 | PmalT_CRPdist-5_rv | ATGACGTTUTTTTATGTGTCTTGTCTGAATTTGCACTGTG TCACAATTCC |
| 5729 | PmalT_CRPdist-6_rv | ATGACGTTUTTTTATGTGTCTGTGTCTGAATTTGCACTGT GTCACAATTCC |
| 5730 | PmalT_CRPdist+4_rv | ATGACGTTUTTTTATGTGAATTTGCACTGTGTCACAATT CC |
| 5731 | PmalT_CRPdist+5_rv | ATGACGTTUTTTTATGGAATTTGCACTGTGTCACAATTCC |
| 5732 | PmalT_CRPdist+6_rv | ATGACGTTUTTTTATGAATTTGCACTGTGTCACAATTCC |
| 5733 | PmalT_RpoDopt0_fw | AAACGTCAUCGCTTGCATTAGAAAGGTTTCTGGCCGACC |
| 5734 | PmalT_RpoDopt35_fw | AAACGTCAUCGCTTGACATAGAAAGGTTTCTGGCCGACC |
| 5735 | PmalT_RpoDopt10_fw | AAACGTCAUCGCTTGCATTAGAAAGGTTTCTGGCCTATAA TATAACCATTAATACGTCGACTCTCGAG |
| 5736 | PmalT_RpoDopt10/35_fw | AAACGTCAUCGCTTGACATAGAAAGGTTTCTGGCCTATAA TATAACCATTAATACGTCGACTCTCGAG |
